# Preparation of Asymmetric Micro-Supercapacitors Based on Laser-Induced Graphene with Regulated Hydrophobicity and Hydrophilicity

**DOI:** 10.3390/nano15080584

**Published:** 2025-04-11

**Authors:** Qing Liu, Wenpeng Wu, Pingping Luo, Hao Yu, Jiaqi Wang, Rui Chen, Yang Zhao

**Affiliations:** Key Laboratory of Cluster Science, Ministry of Education of China, Beijing, Key Laboratory of Photoelectronic/Electrophotonic Conversion Materials, School of Chemistry and Chemical Engineering, Beijing Institute of Technology, Beijing 100081, China; 3120221144@bit.edu.cn (Q.L.); wpwu@bit.edu.cn (W.W.); 3120221150@bit.edu.cn (P.L.); 3220221379@bit.edu.cn (H.Y.); 3120205606@bit.edu.cn (J.W.)

**Keywords:** micro-supercapacitor, laser-induced graphene, regulation of hydrophilicity and hydrophobicity, laser processing, flexible electrode

## Abstract

Asymmetric micro-supercapacitors (AMSCs) with a small size and high energy density can be compatible with portable and wearable electronic devices and are capable of providing stable, long-term power supply, attracting great research interest in recent years. Here, we present a simple and rapid preparation method for AMSCs’ fabrication. By regulating the hydrophilicity and hydrophobicity of coplanar laser-induced graphene (LIG) through the adjustment of the laser parameters, two electrode materials with distinct hydrophilic–hydrophobic properties were selectively deposited by sequentially dip-coating. The LIGs serve as current collectors, with activated carbon and poly (3,4-ethylenedioxythiophene): poly (styrene sulfonate) as active materials. After coating the electrolytes and folding the two electrodes, a high-performance AMSC was achieved. The device exhibits a high areal capacitance of 85.88 mF cm^−2^ at a current density of 0.4 mA cm^−2^, along with an impressive energy density of 11.93 µWh cm^−2^ and a good rate performance. Moreover, it is demonstrated to be highly stable in 500,000 cycles. Two AMSCs in series can supply power to an electronic clock and birthday card. The method of preparing asymmetric electrodes in the same plane greatly facilitates the large-area preparation of AMSCs and series–parallel connection, providing an excellent idea for developing high-performance miniature energy storage devices.

## 1. Introduction

As a promising energy storage technology, micro-supercapacitors exhibit remarkable characteristics, including high power density, fast charge and discharge rates, a small size, maintenance-free, and a long life [1,2,3]. They are highly compatible with the prevailing electronic devices, emphasizing miniaturization, lightweight portability, and high-degree integration [2,4,5,6,7,8,9,10,11,12]. Given these advantages, the development of micro-supercapacitors has become the focus of academic and industrial sectors, and tremendous effort has been devoted to exploring efficient preparation methods [8,13,14,15].

Photolithography, printing, and laser processing technology are mainly used to prepare micro-supercapacitors [11,16,17]. Lithography is mature, has high resolution, and can be processed on various substrates, providing great flexibility in material selection [18]. However, harsh manufacturing conditions are needed, and the manufacturing process is complicated, involving photoresist coating, exposure, development, etching, and photoresist stripping [19,20]. It not only leads to high production costs but also contributes to significant environmental pollution issues, thereby restricting the broader application of this method. The printing method has the advantages of simple operation, easy mastering, and large-area fabrication. The selection of different conductive inks and printing techniques enables the creation of diverse designs for electrode materials and structures on various substrates [21,22,23,24]. However, the printing technique has high requirements for ink, and the uniformity and thickness of the electrode are difficult to control. In addition, the limited printing precision makes it challenging to prepare high-resolution micro-nano structures [25]. In contrast, laser processing technology can accurately realize micro-nano-scale patterning [26], with the advantages of simple operation, high processing speed, high efficiency, and mild conditions, making it widely used in the preparation of micro-supercapacitors [27,28,29].

A laser acting on the surface of specific carbon-based materials such as polyimide will induce a photothermal effect, and the high energy of the laser will be absorbed by the material and converted into heat energy, producing extremely high temperatures on the surface of the material. The high temperature destroys the chemical bond of the base material, and the carbon atoms are rearranged to form a graphene structure, that is, LIG [30]. LIG features the 3-dimensional porous structure resulting from the rapid production of gases during laser irradiation. The chemical composition, morphology, structure, and properties of LIG can be easily tuned by varying the laser parameters. For example, Duy et al. obtained LIGs with different morphologies from sheets to fibres by controlling the pulse-packing density, thus realizing the preparation of LIG fibre forests with millimetre vertical arrangement [31]. Lin et al. investigated the dependence of the sheet resistance of LIG on laser power under environmental conditions and found that the sheet resistance gradually decreased from ~35 Ω per square to ~15 Ω per square as the laser power increased from 2.4 W to 5.4 W [30]. Le et al. discovered that within a certain range, as the laser scanning speed increased, the conductivity of LIG initially increased and then decreased [32]. Li et al. studied the surface wettability of LIG produced in controlled gas environments. They revealed that LIG created in air and oxygen exhibited outstanding hydrophilicity, whereas in argon and hydrogen, it demonstrated remarkable hydrophobicity [33]. Nasser et al. realized the regulation of LIG from hydrophilic to hydrophobic by controlling pulse density in the atmospheric environment [34].

Laser irradiation enables the synthesis and patterning of graphene in one step. The simple preparation and tunability of structure and properties make the LIG widely applicated in energy storage, sensing, catalysis, and separation. Particularly, LIG has excellent physical and chemical properties, such as high conductivity, a large specific surface area, and high porosity, making it possible to be an electrode material for supercapacitors [35,36,37,38]. For example, Peng et al. prepared sandwich-type and planar interdigital flexible supercapacitors by directly using the PI-based LIGs as electrodes, and their area-specific capacitance reached 9.11 mF cm^−2^ at a current density of 0.01 mA cm^−2^ [39]. Nevertheless, bare laser-induced graphene exhibits limitations, such as an insufficient micro-nano structure, limited charge storage sites, and poor wettability, leading to poor energy storage performance, such as specific capacitance and energy density. Li et al. have shown that a symmetric micro-supercapacitor utilizing hydrophilic LIGs as electrodes exhibits an improved specific capacitance of 37 mF cm^−2^, which is attributed to the increased hydrophilicity that facilitates better contact with the electrolyte [33]. However, it is still insufficient for practical application.

Introducing other electrode materials is indispensable for improving the electrochemical performance of micro-supercapacitors based on laser-induced graphene [29,40]. For example, M. Dos S. Klem et al. electrodeposited manganese dioxide (MnO_2_) as a pseudocapacitive material onto a LIG substrate. The resulting LIG/MnO_2_ supercapacitor demonstrated a remarkable area-specific capacitance of 86.9 mF cm^−2^ at a current density of 0.1 mA cm^−2^, which is more than 9-fold of the pristine LIG-based supercapacitor (9.1 mF cm^−2^) [41]. Li et al. significantly enhanced SC performance by electrodepositing pseudocapacitive materials such as polyaniline (PANI) or MnO_2_ onto LIG. For the LIG–MnO_2_ MSC employing a LiCl/PVA electrolyte, a record-high area-specific capacitance of 934 mF cm^−2^ was achieved at 0.5 mA cm^−2^, outperforming the pristine LIG-based MSC (0.8 mF cm^−2^) by over 1160-fold. The LIG–PANI MSC with the H_2_SO_4_/PVA electrolyte demonstrated an area-specific capacitance of 361 mF cm^−2^ at the same current density, surpassing the unmodified LIG MSC (8.4 mF cm^−2^) by 43-fold [42]. Introducing pseudocapacitive materials could significantly improve the capacitance through the electrochemical faradaic reaction. However, they exhibit lower power output and poorer cycling performance than electric double-layer capacitors (EDLCs). Additionally, common methods for introducing the electrode materials, such as electrochemical deposition, in situ growth, and chemical vapour deposition [42,43], need harsh manufacturing conditions and have the limitations of complicated steps, which are tedious and time consuming, significantly reducing production efficiency [40,44].

In this work, we developed a method for the facial and scalable fabrication of high-performance asymmetric micro-supercapacitors. This was accomplished by generating coplanar laser-induced graphene (LIG) characterized by asymmetric hydrophilic and hydrophobic properties, followed by selectively depositing two distinct electrode materials onto each LIG through a wetting–dewetting process. The asymmetric LIGs served as current collectors for the AMSC, while the deposited electrode materials significantly enhanced the energy storage capabilities of LIG-based MSC devices. Due to the programmable procedure for producing LIG current collectors and selectively depositing different active materials in solution, it is an efficient approach for the large-scale production of diverse, flexible AMSC with high performance. To the best of our knowledge, LIG with (super) hydrophilicity/hydrophobicity has been successfully demonstrated to be used in corrosion-resistant coatings, antifreeze surfaces, oil–water separation, and symmetric supercapacitors [45,46,47,48,49]. There are currently no reports addressing the construction of asymmetric supercapacitors by depositing asymmetric electrode materials leveraging the LIGs with asymmetric wetting properties. The fabricated AMSC with H_2_SO_4_/PVA as an electrolyte exhibits a capacitance of 85.88 mF cm^−2^ and an energy density of 11.93 µWh cm^−2^ at a current density of 0.4 mA cm^−2^, which are higher than most of the LIG-based EDLCs. More importantly, it maintains 88.1% of its capacitance after 50,000 cycles, showing excellent long-term stability. Through this fabrication approach, it is easy to implement complex series–parallel connections of AMSCs. Two AMSCs connected in series can stably supply power to an electronic clock and birthday card, demonstrating the great potential for practical application. Such unique characteristics indicate that the laser-assisted regulation of the hydrophilicity and hydrophobicity of LIG provides a practical pathway for constructing high-performance micro-energy storage devices.

## 2. Materials and Methods

### 2.1. Materials

PI tape (0.15 mm) is a commercial product. Activated carbon (AC) was purchased from XFNANO, Nanjing, China. Ketjen Black was used as a conductive carbon black (CB), which was purchased from Duoduo Reagent Co., Suzhou, China. Cyclohexane and polyvinyl alcohol (PVA) were purchased from Aladdin Reagent Inc. All of the above chemicals were used directly without further purification after purchase.

### 2.2. Coplanar Laser-Induced Graphene with Different Hydrophilicity and Hydrophobicity

The preparation of a hydrophilic/hydrophobic LIG current collector by laser at atmosphere: The commercial PI film with a thickness of 0.15 mm was cleaned with anhydrous ethanol and deionized water, and the cleaned PI film was pressed flat on the surface of the glass sheet after drying. After that, the samples were placed in the processing area of the ultraviolet (UV) laser (Beijing LAJAMIN LASER Co., Beijing, China, LM-UVY-5S-Y) for laser processing. By adjusting the laser processing parameters (scanning speed, frequency, processing mode, etc.), rectangular LIG current collectors with hydrophilicity and hydrophobicity (4 mm × 4 mm) were successfully prepared on the same PI film plane. The interval between hydrophilic and hydrophobic LIG current collectors was 4 mm. The processing parameters of hydrophilic/hydrophobic LIG are shown in Table 1.

### 2.3. Preparation of Electrode Materials

#### 2.3.1. Hydrophobic Electrode Material

Activated carbon (the specific surface area is 1800 m^2^/g) and conductive carbon black (Ketjen black) with a mass ratio of 8:1 were added in 6 mL of nonpolar solvent cyclohexane. The mixed solution was magnetically stirred for 30 min and ultrasonically treated for 30 min, making the activated carbon and conductive carbon black fully dispersed in cyclohexane, and the hydrophobic electrode material slurry was obtained.

#### 2.3.2. Hydrophilic Electrode Material

Poly(3,4-ethylenedioxythiophene): poly(styrene sulfonate) (PEDOT:PSS, PH1000, Heraeus, Hanau, Germany) aqueous solution was selected as the hydrophilic electrode material.

### 2.4. Preparation of LIG-Based Electrode

The PI film with hydrophilic and hydrophobic LIGs is immersed in a sequence of hydrophilic electrode material solution and hydrophobic electrode material slurry. After coating with hydrophilic electrode material, the PI film with LIGs is quickly immersed in hydrophobic electrode material slurry before the hydrophilic electrode material is dried. Due to the regulation of hydrophilicity and hydrophobicity, the hydrophilic electrode material will be accurately deposited on the surface of hydrophilic LIG, while the hydrophobic electrode material will be deposited on the surface of hydrophobic LIG and the PI surface of the interval between the two LIGs. After the electrode material is dried, the hydrophobic electrode material deposited on the PI surface of the interval is accurately removed by laser. Finally, hydrophilic and hydrophobic electrodes with a distance of 4 mm were obtained on the same PI film surface, and the dimensions of both electrodes were 4 mm × 4 mm.

For the quantitative study, the specific volume of electrode material solution was used to cover the LIG during the preparation process and evaluate the performance of AMSC accurately. Specifically, 5 μL of hydrophobic electrode material and 40 μL of hydrophilic electrode material were accurately used to prepare two electrodes.

### 2.5. Preparation of PVA/H_2_SO_4_ Hydrogel Electrolyte

Firstly, 6 g of H_2_SO_4_ was slowly dropped into 60 mL of deionized water, and then 6 g of PVA was added to the above solution. The mixture was stirred at 80 °C for 3 h to form a clear and transparent gel solution. After standing at room temperature and naturally cooling down, the PVA/H_2_SO_4_ hydrogel electrolyte was obtained.

### 2.6. Assembly of AMSC

**Figure 1:**
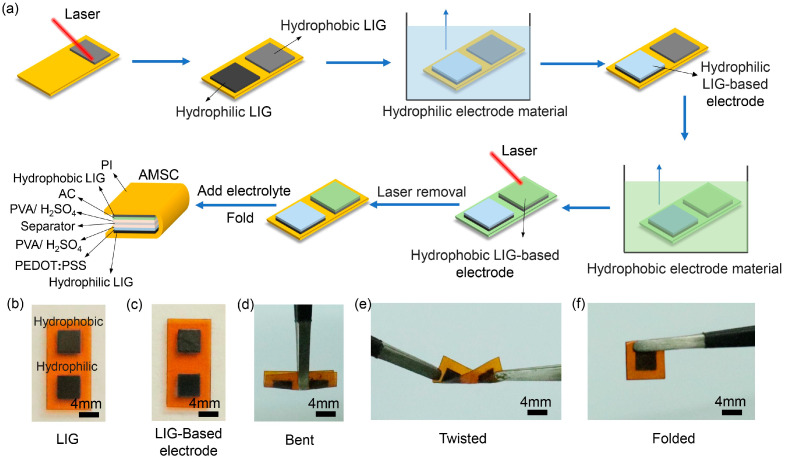
The fabrication and electronic photos of AMSC. (**a**) Schematic diagram of laser-assisted preparation of AMSC; (**b**) hydrophilic and hydrophobic LIG; (**c**) optical image of the LIG covered with electrode materials; (**d**–**f**) LIG-based electrodes at (**d**) bending, (**e**) twisting, and (**f**) folding states.

A piece of PI film with hydrophilic and hydrophobic electrodes was folded along the middle of the interval between the two electrodes. In this process, PVA/H_2_SO_4_ hydrogel electrolyte and a separator (Celgard 3501, 11 Technology Co., Ltd., Changchun, China) were added to the electrode area so that the hydrophilic and hydrophobic electrodes could be closely bonded face to face, without direct contact leading to short circuit. After that, the AMSC based on laser-induced graphene as a current collector was successfully prepared. A schematic illustration of the whole process of preparing AMSC is shown in Figure 1a. For the long-term cycle stability measurement, the AMSC was heat sealed by the polyethylene (PE) film, which can effectively prevent the leakage and evaporation of gel electrolytes and ensure the structural integrity and performance stability of the AMSC.

### 2.7. Characterizations

#### 2.7.1. Electrochemical Measurements

All the electrochemical performance tests were carried out by the CHI 760E Electrochemical Workstation (CH Instruments Inc., Shanghai, China). Cyclic voltammetry (CV), galvanostatic charge–discharge (GCD), and electrochemical impedance spectroscopy (EIS) were used to study the capacitance performance of supercapacitors. The area-specific capacitance $C_{a}$ (F/cm^2^), power density P (Wh/cm^2^), and energy density E (W/cm^2^) of the MSCs can be calculated by the results of the cyclic voltammetry curve and the constant current charge–discharge curve.

The calculation formula of area-specific capacitance is as follows:

(1) $$C_{a}\left(F/{cm}^{2}\right)=\frac{1}{v\times (V_{h}-V_{l})\times A}\int_{V_{i}}^{V_{f}}I(V)dV$$

or

(2) $$C_{a}\left(F/{cm}^{2}\right)=\frac{I\left(A\right)\times ∆t(s)}{∆V\left(V\right)\times A({cm}^{2})}$$

where v is the scanning rate of the CV test (V/s), $V_{h}$ is the highest voltage of the CV test (V), $V_{l}$ is the lowest voltage of the CV test (V), A is the total area of the electrode (cm^2^), and $\int_{V_{i}}^{V_{f}}I\;(V)dV$ is the integrated area of the closed CV curve. I is the charge/discharge current (A), ∆t is the discharge time (s), and ∆V is the voltage range (V).

The energy density E and power density P are calculated as follows:

(3) $$E\;\left(Wh/{cm}^{2}\right)=\frac{I\;(A)*\int_{t_{b}}^{t_{e}}V\;(t)dt}{A\;\left({cm}^{-2}\right)*3600}$$

(4) $$P\left(W/{cm}^{2}\right)=\frac{E\left(Wh/{cm}^{2}\right)}{∆t\;\left(s\right)}\times 3600$$

where I (A) is the charge/discharge current, $t_{b}$ is the time when discharge starts (s), $t_{e}$ is the time when discharge ends (s), $\int_{t_{b}}^{t_{e}}V(t)dt$ is the integral area of the constant current discharge curve, and ∆t is the discharge time (s).

#### 2.7.2. Materials Characterization

The morphology and microstructure were studied by a scanning electron microscope (SEM, JSM-7500F, JEOL, Tokyo, Japan) and optical profilometer (Bruker Contour GT-K 3D, Bruker, Karlsruhe, Germany), and the chemical structure and composition were analyzed by Raman spectroscopy (Horiba LabRAM HR Evolution, HORIBA, Ltd., Kyoto, Japan) and X-ray photoelectron spectroscopy (XPS, Shimadzu/Kratos Axis Supra, Kratos, Manchester, UK). The surface tension tester (Kunshan Shengding SDC 350KS, SINDIN, Dongguan, China) was used to test the contact angle between the LIG surface and water and further calculate its surface energy. The analysis and distribution of elements were tested by an Energy Dispersive Spectrometer (EDS, JSM-7500F, JEOL, Tokyo, Japan). The surface conductivity of LIG was measured by a four-probe resistivity tester (KDY-1, Guangzhou Kunde Technology Co., Ltd. Guangzhou, China). Laser-induced graphene was carried out in the air by a 355 nm ultraviolet laser writing system (LM-UVY-5S-Y, Beijing LAJAMIN LASER Co., Beijing, China).

## 3. Results

### 3.1. Characterization of LIG

Figure 1a schematically illustrates the preparation process of AMSC, including producing hydrophilic and hydrophobic LIG current collectors, coating electrode materials, and assembling a device. Laser parameters have a far-reaching influence on controlling the chemical and physical properties of LIG [30,31]. Two LIGs with distinct hydrophilicity and hydrophobicity were induced on the surface of PI thin films by precisely adjusting the laser parameters (Figure 1b). Electrode materials dispersed in aqueous and nonpolar solutions were selectively deposited on the hydrophilic and hydrophobic LIG surfaces by a wetting–dewetting effect in two-step dipping processes. The resulting electrodes in Figure 1c demonstrate that the active materials were restricted on the respective LIG by the regulation effect of hydrophilicity and hydrophobicity. By virtue of the PI substrate, the electrodes are flexible and can withstand deformations such as bending, twisting, and folding (Figure 1d–f), providing strong support for their application in flexible energy storage.

An ultraviolet (UV) laser (355 nm) was used to synthesize graphene on the PI surface, which is regarded as a combination of photochemical and photothermal processes [50]. Due to the photon energy being of the same order of magnitude as the atomic bonds, the UV laser allows the absorbed photon energy to directly break chemical bonds. Additionally, as the laser fluence increases at shorter wavelengths, the temperature rises due to electron transitions, which further promotes the carbonization of polymers through the photothermal process. Under UV laser irradiation, the chemical bond of the PI is destroyed, and the carbon atoms are rearranged to form a graphene structure.

**Figure 2:**
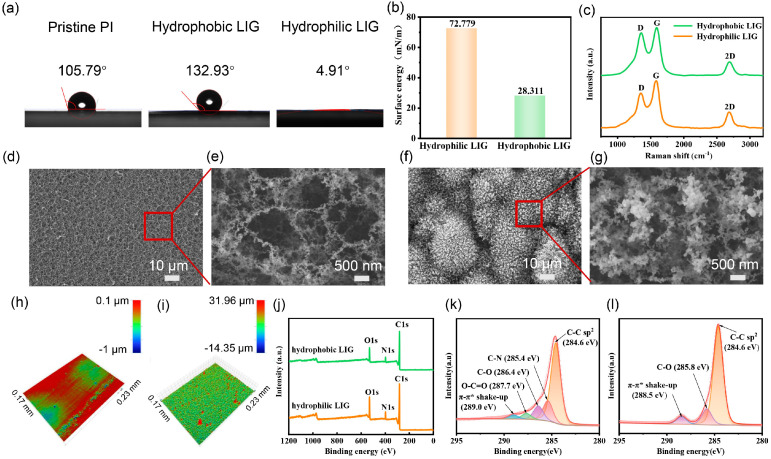
Morphology and structure characterization of LIG. (**a**) The contact angle of pristine PI and hydrophobic and hydrophilic LIGs; (**b**) surface energy and (**c**) Raman spectra of hydrophilic and hydrophobic LIG; (**d**) low magnification and (**e**) high magnification of hydrophilic LIG; (**f**) low magnification and (**g**) high magnification of hydrophobic LIG; surface roughness of (**h**) hydrophilic LIG and (**i**) hydrophobic LIG; (**j**) the XPS survey spectra of LIGs; The XPS C1s spectra of (**k**) hydrophobic and (**l**) hydrophilic LIGs.

During the laser process, a series of LIGs were successfully prepared on the surface of PI thin films with a thickness of 150 μm by precisely adjusting the key parameters such as laser scanning speed, frequency, and repeat times. The hydrophobic LIG was produced by using single-laser irradiation. When the UV laser is acting on the PI film, it rapidly breaks the chemical bonds and forms graphene on the surface of the PI film. For the hydrophilic LIG, double irradiation processes were applied. The first laser irradiation formed the LIG base layer, followed by the secondary laser passing in the perpendicular direction, altering the morphology and chemical structure of the surface layer and resulting in hydrophilic LIG [51]. As shown in Figure S1, when using a single-laser scan, the LIGs show hydrophobicity with contact angles ranging from 90.5° to 132.8° in the 70–80 kHz frequency range. After a secondary laser scan perpendicular to the first scan, the LIGs show hydrophilicity with decreased contact angles (4.9–41.3°) in the 70–80 kHz frequency range. This indicates that hydrophilicity mainly originates from the second laser scan. The hydrophobic LIG shows a maximum contact angle of 132.93° at 72 kHz, and the hydrophilic LIG shows a minimum contact angle of 4.91° at 74 kHz (Figure 2a).

In terms of electrical property (Figure S2), the sheet resistance of hydrophobic LIGs slightly decreased with the decrease in the laser frequency, which can be attributed to the increased carbonization degree of the PI precursor. For the hydrophilic LIG produced by double-laser irradiation, the laser parameter had a negligible effect on the sheet resistance. At laser frequencies of 72 kHz (hydrophobic) and 74 kHz (hydrophilic), the LIGs also showed a low sheet resistance of 47.03 Ω/sq and 34.03 Ω/sq. Based on the comprehensive consideration of the contact angle and resistance, the optimal parameters for preparing hydrophilic (4.91°) and hydrophobic (132.93°) LIG were determined and are displayed in Table 1. If not specified, LIGs studied in this work were produced under the optimal parameters.

A surface tension tester, scanning electron microscope (SEM), Raman spectroscopy, and X-ray photoelectron spectroscopy (XPS) were used to characterize the properties of LIGs. The surface energy of hydrophobic and hydrophilic LIGs was measured to be 28.31 mN/m and 72.78 mN/m, respectively (Figure 2b), which is consistent with the results of the contact angle. The high surface energy of hydrophilic LIG means a strong interaction between LIG surface molecules and water molecules, leading to the easy spreading of water molecules on the LIG surface, exhibiting excellent hydrophilicity. In contrast, hydrophobic LIG has low surface energy and a weak intermolecular force, making it difficult for the surface to be wetted by water molecules and showing excellent hydrophobicity. The remarkable difference in hydrophilicity and hydrophobicity of the LIG surface achieved by laser treatment provides solid and reliable support for the subsequent selective deposition of hydrophilic and hydrophobic electrode materials on each LIG surface.

In the Raman spectra of LIGs (Figure 2c), three intensive peaks corresponding to the characteristic peaks of graphene can be observed, demonstrating the successful preparation of LIG. The D peak at about 1350 cm^−1^ comes from the defects or bent sp^2^ carbon bonds, the G peak at about 1580 cm^−1^ originates from the tensile vibration between SP carbon atoms, and the 2D peak at about 2700 cm^−1^ comes from the second-order region boundary phonons [30]. The presence of a 2D peak implies the existence of single-layer graphene sheets [30]. The intensity ratio of the D peak and the G peak (I_D_/I_G_) is a measure of disorder and defect structure in graphene [30,52]. The I_D_/I_G_ ratio of hydrophobic LIG and hydrophilic LIG is 0.83 and 0.67, respectively, indicating that hydrophilic LIG has reduced defect structures and an improved order of materials. That is the reason why hydrophilic LIG has a higher electrical conductivity (Figure S2), which is strongly associated with the order of graphene. The higher I_D_/I_G_ can be attributed to the fact that hydrophilic LIG has undergone one more laser treatment in the vertical direction relative to hydrophobic LIG. Repeated laser treatment has been shown to enhance the graphitization process, reduce the defects, and improve the overall quality of graphene [53,54,55].

The microstructure and surface morphology of LIGs were studied by SEM (Figure 2d–g). It can be observed that there are significant differences in morphology and microstructure between hydrophilic and hydrophobic LIGs. Figure 2d,e show that the hydrophilic LIG surface presents a dense porous structure caused by the release of small molecular gas from PI under laser action [30,56]. The highly uniform reticular porous microstructure could significantly increase the contact area between the material surface and water and provide more contact sites and channels for water molecules to access. Simultaneously, its high surface energy enhances the interaction with water molecules, resulting in the fast absorption and spreading of water, accounting for excellent hydrophilicity [57]. In sharp contrast, a large number of micron-scale protrusions are distributed on the surface of hydrophobic LIG (Figure 2f,g). These protrusions hinder the spreading of water on the surface by their micro-scale geometry, which reduces the actual contact area between water and the solid surface. In addition, the air is easily trapped in the concave region of the rough surface, forming an air layer. These air layers will create a stable liquid–gas interface after contact with water, reducing direct contact between water and the solid surface [52]. Moreover, the low surface energy further weakens the affinity between water molecules and the surface. The synergistic effect of the two makes the LIG exhibit excellent hydrophobicity [58].

The three-dimensional optical profiles are also consistent with the results of SEM. As shown in Figure 2h,i, the surface roughness of hydrophilic LIG (0.005 μm) is three orders of magnitude lower than that of hydrophobic LIG (1.332 μm). The remarkable difference in surface roughness between the two LIGs reflects that LIGs with different hydrophilicity and hydrophobicity prepared under different laser parameters experience different physical and chemical transformations, which leads to the difference in the macroscopic properties of materials.

XPS was carried out to study the reasons for LIG showing different hydrophilicity and hydrophobicity from the chemical structure in depth. The survey spectra in Figure 2j show that both hydrophilic and hydrophobic LIGs contain a large amount of carbon and oxygen and a small amount of nitrogen. The element contents are summarized in Table 2. It shows that the hydrophilic LIG has an increased C content and reduced N content, which can be ascribed to the enhanced graphitization caused by double-laser irradiation. The C1s peak of hydrophobic LIG (Figure 2k) can be deconvolved into C–C sp^2^ (ca. 284.6 eV), C–N (ca. 285.4 eV), C–O (ca. 286.4 eV), O-C=O (ca. 287.7 eV), and π-π* shake-up (ca. 289.0 eV). The C1s peak of hydrophilic LIG (Figure 2l) can be deconvolved into C–C sp^2^ (ca. 284.6 eV), C–O (ca. 286.5 eV), and π-π* shake-up (ca. 289.1 eV) [59,60,61], where the C-N and O-C=O are negligible. The contents of various chemical bonds obtained from C1s spectra are displayed in Table 3. The content of different chemical bonds in LIG plays a vital role in its wetting properties [59]. The C-O bond and the O-C=O bond can effectively promote the hydrophilicity of materials through hydrogen bonding and electrostatic interaction with water molecules [62]. Notably, the C-O bond is more dominant in the hydrophilicity due to the formation of C-OH. Table 2 and Table 3 clearly show that the hydrophilic LIG has a higher content of the O element (17%) and C-O groups (13.8%), enabling it to interact strongly with water molecules and thus showing excellent hydrophilicity. On the contrary, the lower content of the O element (12.7%) and the C-O groups (11.4%) of hydrophobic LIG makes it have a weaker interaction with water molecules, showing more significant hydrophobicity.

### 3.2. Characterization of LIG-Based Electrode

**Figure 3:**
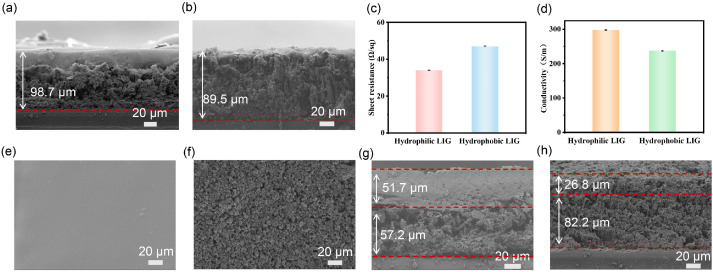
Characterization of LIGs and LIG-based electrodes. The cross-sectional SEM images of (**a**) hydrophilic LIG and (**b**) hydrophobic LIG; (**c**) average sheet resistance and (**d**) conductivity of LIGs; SEM images of (**e**) hydrophilic LIG-based electrode and (**f**) hydrophobic LIG-based electrode; the cross-sectional SEM images of (**g**) hydrophilic LIG-based PEDOT:PSS electrode and (**h**) hydrophobic LIG-based AC electrode.

The current collector plays an indispensable key role in the micro-supercapacitor. High conductivity is essential for fast and efficient charge transfer, which is of great importance to the charging and discharging efficiency and overall performance of the supercapacitor [63]. To evaluate the suitability of hydrophilic and hydrophobic LIGs as MSC current collectors, the sheet resistance of LIGs was measured via the four-probe method, and the conductivity was calculated based on the sheet resistance and thickness evaluated from the cross-sectional SEM images in Figure 3a,b. The average sheet resistance of hydrophilic and hydrophobic LIGs is 34.03 Ω/sq and 47.03 Ω/sq (Figure 3c), respectively, corresponding to average conductivities of 298 S/m and 238 S/m (Figure 3d). The high conductivity proves its feasibility as an MSC current collector, which lays a solid foundation for the further research and development of high-performance MSC based on LIG.

Subsequently, corresponding hydrophilic and hydrophobic active materials were selectively covered on the hydrophilic and hydrophobic LIGs by a rapid two-step dipping process to prepare the asymmetric electrodes of AMSC. For AMSC, it is significant to balance the amount of charges stored in the positive and negative electrodes to improve performance and prolong its service life [64,65]. For quantitative analysis, electrodes with varied mass loading were prepared by dropping a constant volume of hydrophobic slurry with varied AC concentrations onto the hydrophobic LIGs and assembled into symmetric MSCs. The electrochemical properties of the symmetric MSCs were studied via CV and GCD, as shown in Figure S3. At a current density of 1 mA cm^−2^, the areal capacitance of symmetric MSC increased first and then decreased with the increase in AC loading. When the AC loading was 1.25 mg cm^−2^, the MSC showed the highest area capacitance of 61.30 mF cm^−2^. This indicates that an AC loading of 1.25 mg cm^−2^ is appropriate and that the thickness of the electrode is moderate, which can ensure effective contact between the electrode and electrolyte and provide a better ion transmission path. In addition, in the process of full contact with the electrolyte, the electrode can maintain good stability without surface shedding, thus having better electrochemical performance.

After that, the hydrophobic electrode was prepared with an AC loading of 1.25 mg cm^−2^ and used as the positive electrode. Different volumes of hydrophilic electrode material (PEDOT:PSS) solution were added to prepare hydrophilic electrodes, which were used as negative electrodes of AMSC. Similarly, CV and GCD tests were carried out on AMSCs, and the area capacitance was calculated. As shown in Figure S4, when the volume of PEDOT:PSS is 10, 20, 30, 40, 50, and 60 μL, the area capacitance of AMSCs is 30.30, 39.30, 63.10, 78.00, 77.10, and 69.60 mF cm^−2^, respectively. The prepared AMSC exhibits a maximum area capacitance of 78.00 mF cm^−2^ at 40 μL, which implies that the PEDOT:PSS has a sufficient active substance and appropriate thickness, contributing to matched active sites and high ion/electron transmission efficiency. Therefore, it was selected to serve as a negative electrode for AMSC devices.

Based on the hydrophilic and hydrophobic LIGs, the SEM images of the two electrodes with an optimal mass loading of electrode materials are shown in Figure 3e–h. The PEDOT:PSS electrode is featureless with uniform and smooth surface morphology (Figure 3e). In contrast, the activated carbon shows a granular structure and rough appearance (Figure 3f). Compared to the bare LIGs, an apparent double-layer structure was shown after depositing active materials (Figure 3g,h). The thickness of the electrode layer can be estimated to be 51.7 μm for the PEDOT:PSS layer and 26.8 μm for the AC layer. Both the upper electrode layers are closely bonded with LIG layers, which will facilitate the electron transportation between the electrodes and current collectors. This can be attributed to the similar surface properties between the LIG and deposited electrode materials.

### 3.3. Electrochemical Properties of AMSC

**Figure 4:**
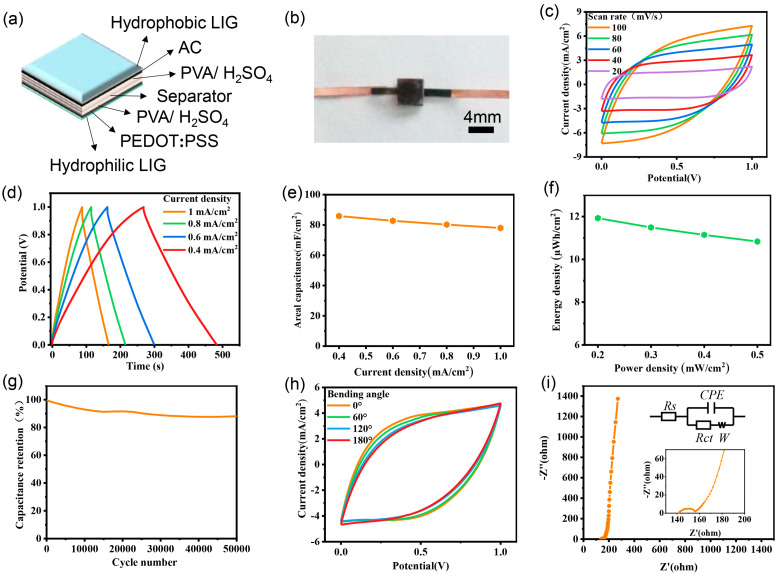
Electrochemical properties of a single AMSC. (**a**) Device structure and (**b**) optical photos of AMSC; (**c**) CV curves at different scanning speeds; (**d**) GCD curves at different current densities; (**e**) area capacitance at various current densities calculated from GCD curves; (**f**) energy density and power density of AMSC; (**g**) capacitance retention of AMSC during 50,000 cycles; (**h**) CV curves at different bending angles; (**i**) Nyquist plot of AMSC with an enlarged view and equivalent circuit diagram in the insert.

Using an activated carbon electrode as the positive electrode and the PEDOT:PSS electrode as the negative electrode, adding PVA/H_2_SO_4_ hydrogel electrolyte and folding, a sandwiched AMSC was successfully prepared. The device structure and optical picture of the AMSC are shown in Figure 4a and Figure 4b, respectively. Figure 4c shows the CV curve of AMSC with a scan rate increasing from 20 mV s^−1^ to 100 mV s^−1^ in the voltage range of 0~1 V. When the scanning speed is 20 mV s^−1^, the shape of the CV curve is close to a rectangle, which suggests that the adsorption and desorption of electrolyte ions are sufficient during the charging and discharging process, indicating the typical electric double layer capacitance behaviour of AMSC. In addition, the GCD curves of AMSC at different current densities are presented as a symmetrical triangle (Figure 4d), which shows that the supercapacitor has good reversibility during the charge–discharge process, further demonstrating the typical electric double-layer capacitive performance. The areal capacitance calculated according to GCD curves at different current densities is shown in Figure 4e. The areal capacitance (Ca) of AMSC reaches 85.88 mF cm^−2^ at a current density of 0.4 mA cm^−2^, which is competitive compared with other LIG-based MSCs in Table S1 [39,42,43,66,67,68,69,70,71,72]. Moreover, even when the current density increased to 1 mA cm^−2^, the areal capacitance of AMSC remained at 78 mF cm^−2^, indicating excellent rate performance. The energy density and power density of AMSC are shown in Figure 4f. When the power densities are 0.2 and 0.4 mW cm^−2^, the energy densities are 11.93 and 10.83 µWh cm^−2^, respectively. The long-term cycle stability of AMSC is further evaluated and shown in Figure 4g. After 50,000 charge–discharge cycles, AMSC maintains its 88.1% capacitance, showing good cycle stability.

In addition, the AMSC shows stable energy storage performance under deformations. As presented in Figure 4h, the CV curves of AMSC at the bending angles of 0°, 60°, 120°, and 180° exhibit negligible changes. When the bending angle is up to 180°, the capacitance of AMSC maintains 87% of its initial value, indicating that the AMSC device is highly flexible to withstand bending in an extensive range of angles without significant capacitance decay. It enables AMSC devices to meet the needs of flexible electronic devices, significantly expanding their application in flexible electronics and wearable devices.

It is worth noting that the areal capacitance of the symmetrical MSC based on hydrophilic electrode material and hydrophobic electrode material is only 72.00 mF cm^−2^ and 72.84 mF cm^−2^ at the current density of 0.4 mA cm^−2^ (Figures S5 and S6), respectively. The AMSC shows an increased capacitance compared with the corresponding symmetrical MSCs. The reason is that AMSC with AC as the positive electrode and PEDOT:PSS as the negative electrode can fully integrate and give full play to the unique advantages of AC and PEDOT:PSS, thus achieving a synergistic effect and improving the overall electrochemical performance. AC provides a large number of charge storage sites, while PEDOT:PSS ensures good electron conduction and faster ion transport. The synergistic effect of the two electrode materials enables the whole supercapacitor to store and release charges more efficiently, thus improving the areal specific capacitance [73,74,75].

The kinetics of ion transport for the AMSC was studied by EIS with a frequency range from 100 kHz to 0.01 Hz (Figure 4i). The Nyquist plot for the AMSC shows a semicircle in the high-frequency range and an inclined straight line in the low-frequency range. The inclined straight line in the low-frequency region is closely related to the capacitance behaviour. The large slope of the straight line indicates a near-ideal capacitive behaviour with fast ion diffusion [43,76]. The equivalent solution resistance (Rs) is 141.6 Ω and the charge transfer resistance (Rct) is 15.2 Ω, which indicates the rapid ion diffusion and charge transfer between electrode and electrolyte [77]. The Rs and Rct values of AMSC fall between those of symmetrical MSC based on hydrophilic electrode material (Rs and Rct are 92.8 Ω and 6.8 Ω, respectively, Figure S6) and hydrophobic electrode material (Rs and Rct are 168.5 Ω and 15.62 Ω, respectively, Figure S5), indicating the efficient charge transfer within the electrode and highlighting the combined effect of the two electrodes.

**Figure 5:**
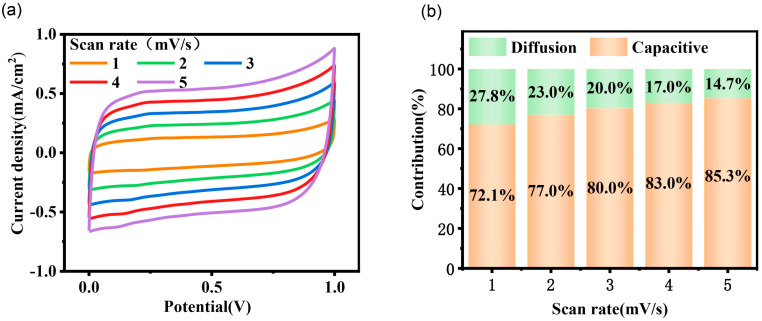
Capacitance contribution of AMSC. (**a**) CV curve of AMSC and (**b**) corresponding capacitance contribution rate at different scanning speeds.

The CV curves of the AMSC are additionally analyzed to investigate the charge storage mechanism according to Dunn’s method. As shown in Figure 5, as the scan rate increases, the contribution of the capacitive-controlled process to the total capacity increases. At a scan rate of 5 mV s^−1^, the capacitive-controlled process accounts for 85.3% of the total capacity. These results demonstrate the capacitive-dominant characteristics and rapid kinetics of the AMSC devices, indicating that forming an electric double layer on the surface of the electrode materials is the primary mechanism for charge storage.

### 3.4. Application of AMSC

**Figure 6:**
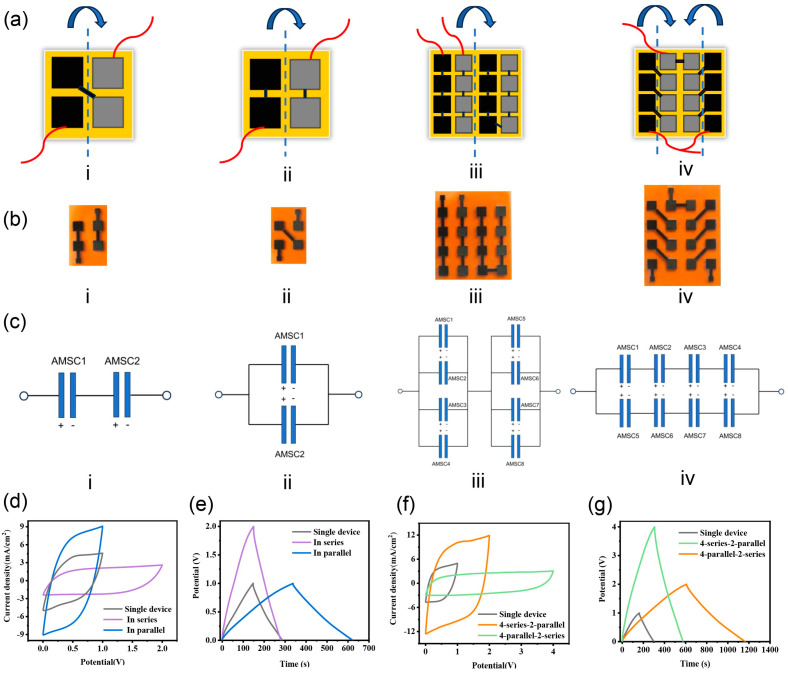
Demonstration of different series–parallel combinations of AMSCs. (**a**) Schematic diagram, (**b**) physical diagram, and (**c**) circuit diagram of different series–parallel connection modes: two AMSCs (i) in series and (ii) in parallel connections and (iii,iv) series–parallel combinations; (**d**–**g**) electrochemical performance of the series–parallel-connected AMSCs.

Practically, it is difficult for a single AMSC to satisfy specific energy and power needs. Various series–parallel-connected AMSCs are preferable for practical applications. Reliable connections are essential to construct the AMSC integration system. The highly conductive LIG can also serve as the electroconductive connector between AMSC devices and greatly simplify the integration process, as demonstrated in Figure 6. Through the rational design of LIG patterns (Figure 6a), AMSCs integrated in series, parallel, and series–parallel connections can be realized (Figure 6b,c). More importantly, the electrochemical performance of integrated AMSCs connected by LIG is comparable to that connected by copper foil (Figure S7), demonstrating the reliable connectivity of LIG. The CV and GCD curves of the integrated devices based on LIG connectors are displayed in Figure 6d–g, showing that the series–parallel connection modes have a significant effect on the electrochemical performance of integrated devices. In a series configuration, the voltage increased almost proportionally to the number of AMSCs while the discharging time remained constant. In a parallel configuration, the current and capacitance increase nearly proportional to the number of AMSCs with the voltage unchanged. It suggests that the AMSCs show ideal series–parallel characteristics.

**Figure 7:**
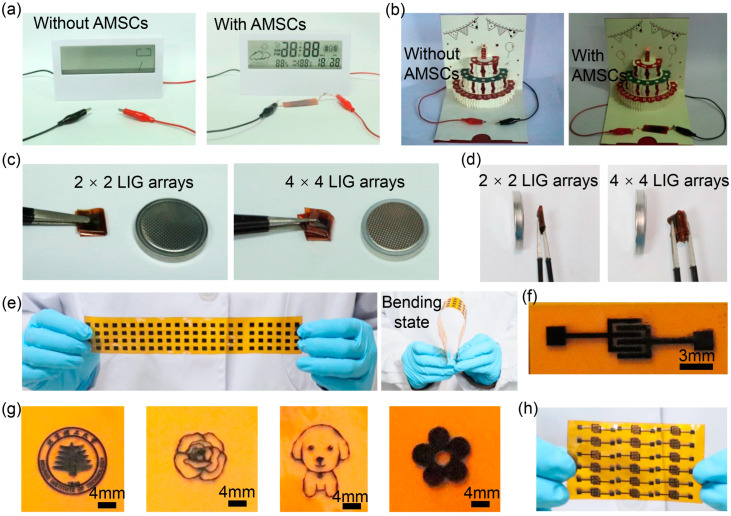
Applications of AMSCs. Supply power to (**a**) an electronic clock and (**b**) a birthday card using two AMSCs connected in series; AMSCS with 2 × 2 and 4 × 4 arrays can be folded to be (**c**) smaller and (**d**) thinner than a button cell; (**e**) optical pictures of hydrophilic and hydrophobic LIG arrays prepared on a large scale; optical photos of (**f**) interdigitated LIG and (**g**) various LIG patterns; (**h**) optical pictures of planar interdigital AMSCs on a large scale.

Due to the good energy storage performance and series–parallel characteristic, two AMSCs connected in series can provide stable power support to an electronic clock and birthday card (Figure 7a,b), which fully demonstrates the feasibility and effectiveness of AMSCs in supplying energy for small electronic devices and provides a preliminary basis for its application in practical scenes. In addition, the inherent flexibility of AMSCs and PI film allows the integrated AMSCs with series–parallel connections to be folded, effectively reducing the occupied area. As shown in Figure 7c,d, the size of the whole device based on 2 × 2 and 4 × 4 LIG arrays is much smaller than a typical button cell after folding, making it favourable for the application of AMSCs in microelectronic systems as micro-flexible power units.

Moreover, the developed preparation method is simple and does not involve complicated processes and harsh conditions. It can be easily transferred to the large-scale fabrication of various flexible AMSCs. Figure 7e displays a large-scale electrode array (4 × 24) on flexible PI film fabricated by this method, with hydrophilic and hydrophobic electrodes alternatively distributed. Through the design of graphics, various LIG patterns can be obtained after laser processing. A few examples are displayed in Figure 7f,g, such as an interdigital electrode, a university badge, and a flower. Significantly, despite the AMSCs with a sandwich structure assembled by folding two electrodes, planar interdigital AMSCs can also be easily achieved by preparing interdigital LIGs (interdigital length is 3 mm, interdigital width is 0.5 mm, and spacing is 0.25 mm), as shown in Figure 7f. Through the same technical path, it is expected to realize the efficient preparation of large-scale planar interdigital AMSCs (Figure 7h). It demonstrates the flexibility and adaptability of this preparation method in creating desirable structures and mass production, providing a broader prospect for the diversified application of AMSCs.

## 4. Discussion

The laser process has a significant influence on the structure (morphology and chemical structure) and properties (wetting property and electrical conductivity) of LIGs, thus, the electrochemical performance of the LIG-based micro-supercapacitors. The hydrophobic LIG is produced by single-laser irradiation. With the increased laser energy, the LIG shows increased conductivity (Figure S2) and hydrophobicity (Figure S1), which can be attributed to the increased carbonization degree of the PI precursor. The hydrophilic LIG is produced via a perpendicular double-laser irradiation. The first laser irradiation produced an LIG base layer and the second further modified the structure of the surface layer. A double-laser pass leads to a higher graphitization and conductivity of LIG, which is evidenced by the decreased ID/IG ratio (Figure 2c) and more predominant C-C sp^2^ bonds (Figure 2l). More importantly, it shows a dense porous structure (Figure 2d,e) with a higher content of the O element and C-O groups (Table 3), which has a large contact area and a strong affinity with water molecules, contributing to the strong hydrophilicity.

Due to the asymmetric hydrophilicity/hydrophobicity of LIGs on the PI surface, hydrophilic PEDOT:PSS and hydrophobic AC, which are dispersed in water and nonpolar cyclohexane, can be quickly and selectively deposited on each LIG surface, respectively, via the wetting–dewetting effect during two-step dip-coating. The cross-sectional images of the electrodes (Figure 3g,h) show that both the upper electrode layers are closely bonded with LIG layers, which is advantageous for facilitating electron transportation between the electrodes and current collectors. Due to the programmable procedure for producing LIG current collectors and selectively depositing active materials in solution, this method has great potential to construct various flexible AMSCs on a large scale, as demonstrated in Figure 7e–h.

Compared to the symmetric MSC, the AMSC constructed by the AC positive electrode and PEDOT:PSS negative electrode shows an enhanced capacitance. It indicates that the AMSC device can fully integrate and give full play to the unique advantages of AC and PEDOT:PSS electrodes, thus achieving a synergistic effect and improving the overall electrochemical performance. According to the EIS plot (Figures S5c and S6c), the Rs and Rct of the hydrophilic symmetric MSC based on PEDOT:PSS are 92.8 Ω and 6.8 Ω, respectively, while those of the hydrophobic symmetric MSC based on AC are 169.5 Ω and 15.62 Ω, respectively. AC is a type of EDLC material with high specific surface area; however, the hydrophobic nature results in inadequate wetting with the aqueous electrolyte, hindering efficient ion transport and charge storage. In contrast, PEDOT:PSS exhibits strong hydrophilicity, which facilitates the absorption of electrolytes and consequently enhances ion adsorption. The Rs and Rct of the overall AMSC are 141.6 Ω and 15.2 Ω (Figure 4i), indicating the efficient charge transfer within the electrode, ascribing to the combination effect of the two electrodes. The charge storage mechanism study also demonstrates the capacitive-dominant characteristics and rapid kinetics of the AMSC devices.

The fabricated AMSC shows a high capacitance of 85.5 mF cm^−2^ at a current density of 0.4 mA cm^−2^, allowing the practical application of powering electronic clocks and birthday cards. Additionally, the AMSC exhibits excellent long-term cycle stability, maintaining 88.1% of the capacitance after 50,000 charging–discharging cycles. These results demonstrate that the established method of creating LIG current collectors with asymmetric hydrophilicity/hydrophobicity and selectively depositing asymmetric electrode materials leveraging the wetting–dewetting effect presents a strong candidate for achieving high-performance micro-energy storage devices with asymmetric configuration.

## 5. Conclusions

In summary, a facial and scalable method was successfully demonstrated to fabricate high-performance AMSC. By regulating the laser parameters, LIGs with a high contact angle (132.9°) and a low contact angle (4.9°) were obtained to be the current collectors of AMSCs. Different active electrode materials were selectively deposited on each LIG by the regulating effect of hydrophilicity and hydrophobicity. The prepared sandwiched AMSC based on the AC positive electrode and the PEDOT:PSS negative electrode shows excellent energy storage capacity with a high area capacitance of 85.88 mF cm^−2^ at a current density of 0.4 mA cm^−2^, which is significantly higher than many reported MSCs based on LIG. Two AMSCs connected in series can stably supply power to small electronic devices such as electronic clocks and birthday cards, verifying their potential in practical applications. Moreover, the AMSC has excellent flexibility and shows stable electrochemical performance under deformations. This method allows for the large-scale fabrication of flexible AMSCs with various configurations, which will further facilitate the broad application of AMSCs in miniaturized electronic integration systems.

## Figures and Tables

**Table 1:** Laser processing parameters for preparing hydrophilic/hydrophobic current collectors.

	Hydrophobic LIG	Hydrophilic LIG
Focal length (cm)	26.15	26.15
Scanning rate (mm/s)	10	10
Frequency (kHz)	72	74
Filling method	Horizontal direction	Horizontal + vertical direction
Line spacing (mm)	0.01	0.01

**Table 2:** Element contents of hydrophobic and hydrophilic LIG obtained by XPS.

	Hydrophobic LIG	Hydrophilic LIG
C	80.9%	81.3%
N	6.4%	1.7%
O	12.7%	17.0%

**Table 3:** Chemical bond contents of hydrophobic and hydrophilic LIGs obtained from XPS C1s spectrum.

	Hydrophobic LIG	Hydrophilic LIG
C-C sp^2^	64.7%	79.8%
C-N	15.5%	/
C-O	11.5%	13.8%
O-C=O	4.9%	/
π-π* shake-up	3.5%	6.4%

## Data Availability

The original contributions presented in this study are included in the article/Supplementary Materials. Further inquiries can be directed to the corresponding authors.

## Supplementary Materials

The following supporting information can be downloaded at: https://www.mdpi.com/article/10.3390/nano15080584/s1.
