# Supplementary material for: Preparation of Asymmetric Micro-Supercapacitors Based on Laser-Induced Graphene with Regulated Hydrophobicity and Hydrophilicity
Source: Nanomaterials (Basel). 2025 Apr 11;15(8):584. doi: 10.3390/nano15080584 (PMC12029501; doi:10.3390/nano15080584)
Supplement: Supplementary file 1 [file nanomaterials-15-00584-s001.zip › nanomaterials-3532843-supplementary.pdf]

Preparation of Asymmetric Micro Supercapacitors Based on Laser-Induced Graphene with Regulated Hydrophobicity and Hydrophilicity

Qing Liu, Wenpeng Wu, Pingping Luo, Hao Yu, Jiaqi Wang, Rui Chen*, Yang Zhao*

Key Laboratory of Cluster Science, Ministry of Education of China, Beijing, Key Laboratory of Photoelectronic/Electrophotonic Conversion Materials, School of Chemistry and Chemical Engineering, Beijing Institute of Technology, Beijing 100081, P. R. China.

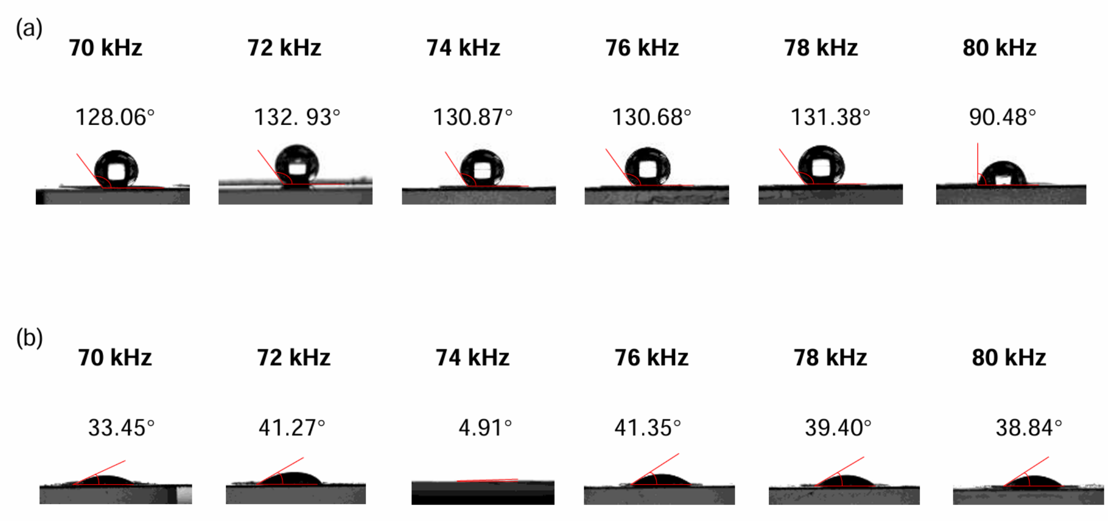

Figure S1. Contact angle of LIGs corresponding to laser processing frequencies of 70, 72, 74, 76, 78 and 80 kHz. (a) The contact angles of hydrophobic LIG obtained at different laser frequencies when the filling method is once in the horizontal direction; (b) The contact angles of hydrophilic LIG obtained at different laser frequencies when the filling method is once in both horizontal and vertical directions.

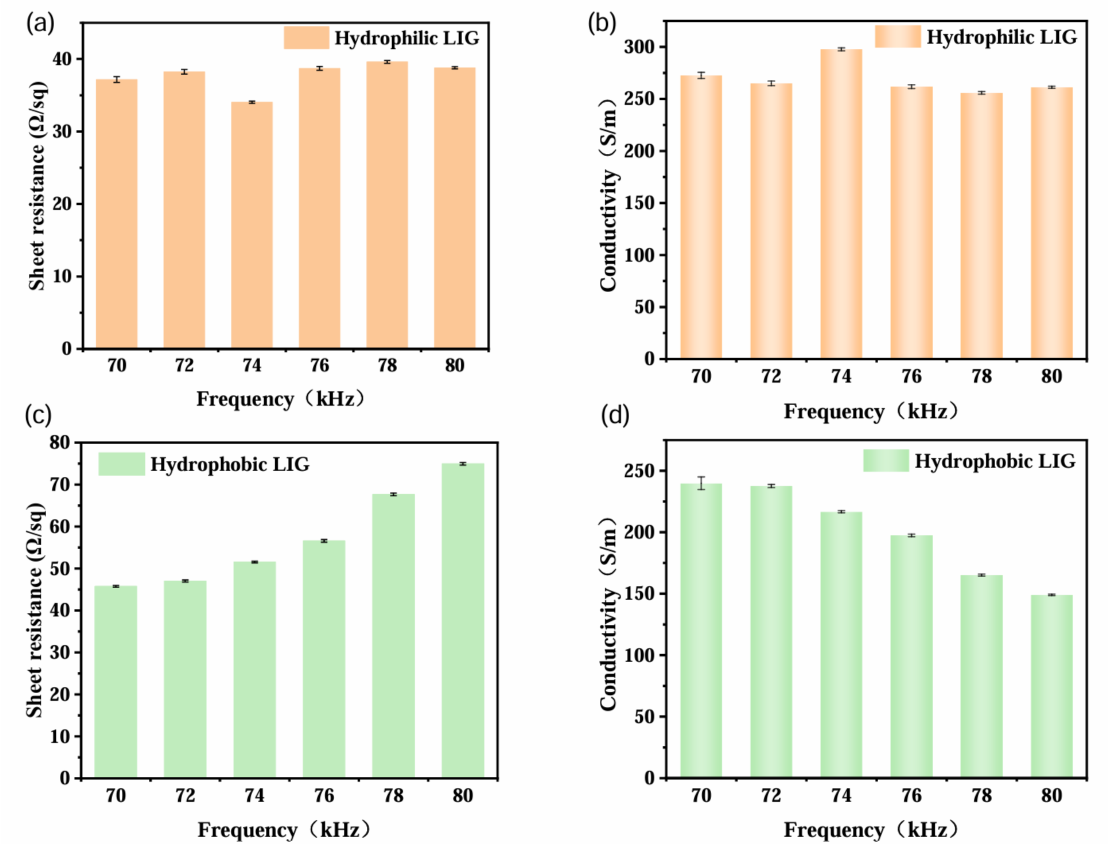

Figure S2. The electrical conductive property of LIG obtained at different laser processing frequencies. (a) Average sheet resistance and (b) conductivity of hydrophilic LIG; (c) Average sheet resistance and (d) conductivity of hydrophobic LIG.

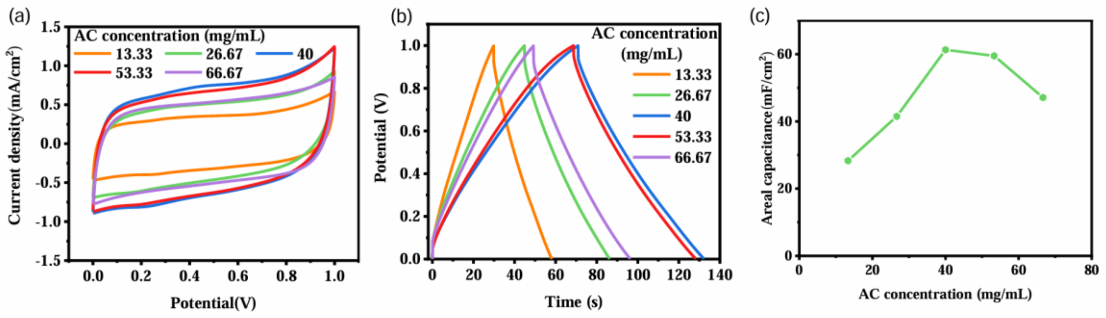

Figure S3. The electrochemical performance of symmetric MSCs. (a) CV curves, (b) GCD curves and (c) the calculated area capacitance of symmetric MSCs with varied AC loading.

Electrodes with varied mass loading were prepared by dropping a constant volume of hydrophobic slurry with varied AC concentrations onto the hydrophobic LIG, and assembled into MSC. In detail, A series of hydrophobic electrode material slurries with different AC concentrations were prepared, which were 13.33 mg mL^{-1}, 26.67 mg mL^{-1}, 40 mg mL^{-1}, 53.33 mg mL^{-1} and 66.67 mg mL^{-1}, respectively. Then, symmetrical MSCs with various mass loadings of active materials were prepared by dropping constant volume (5 μL) of hydrophobic electrode material slurries in different concentrations. At a current density of 1 mA cm^{-2}, the symmetric MSCs show the maximum area specific capacitance at AC concentration of

40 mg mL^{-1}. It is determined to be the optimal condition and the AC loading is calculated to be 1.25 mg cm^{-2}.

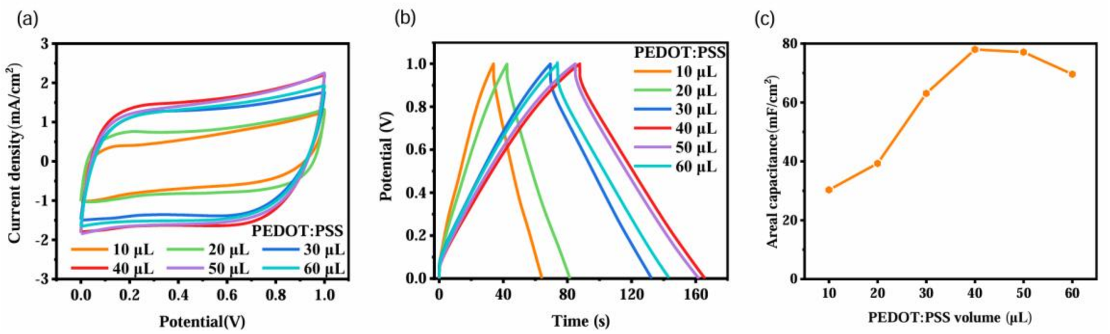

Figure S4. The electrochemical performance of AMSCs with varied PEDOT:PSS loading. (a) CV curves, (b) GCD curves and (c) the calculated area capacitance of the AMSCs.

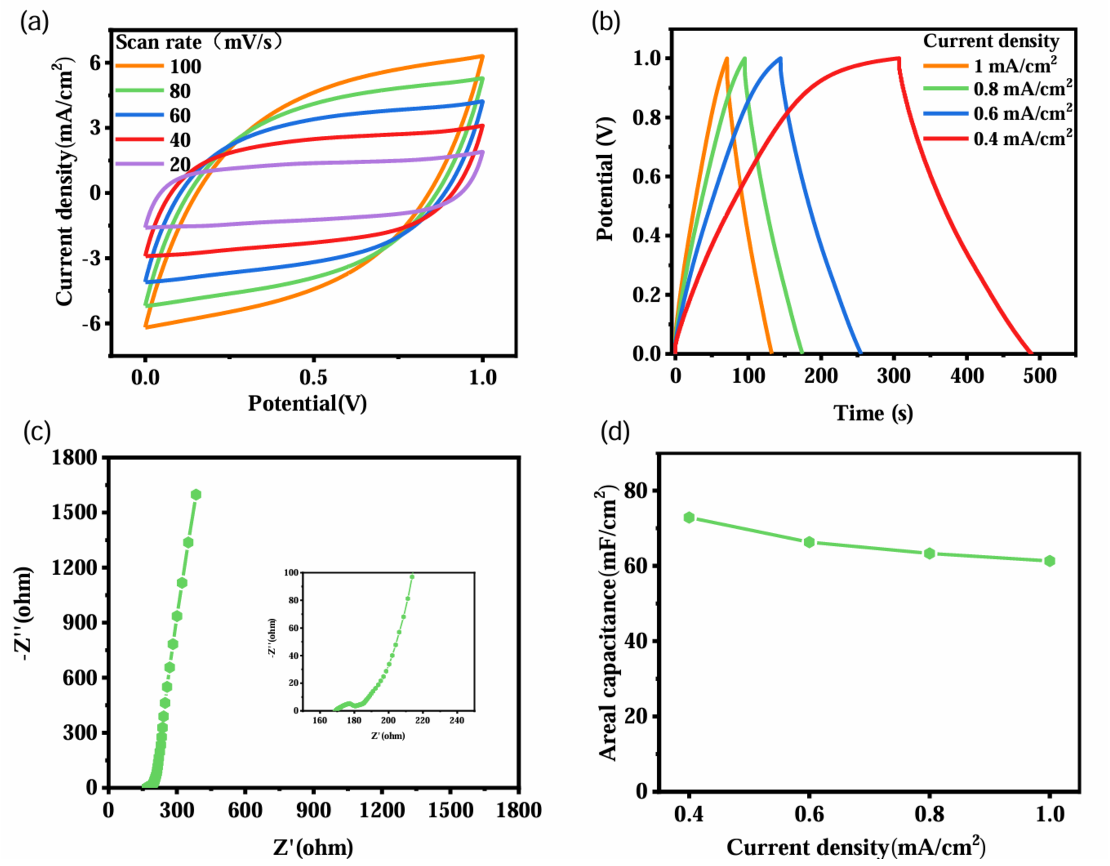

Figure S5. The electrochemical performance of symmetrical MSC based on AC electrode. (a) CV curves at different scanning speeds; (b) GCD curves at different current densities; (c) Nyquist plot with an enlarged view in the insert; (d) Area capacitance at various current densities calculated from GCD curves.

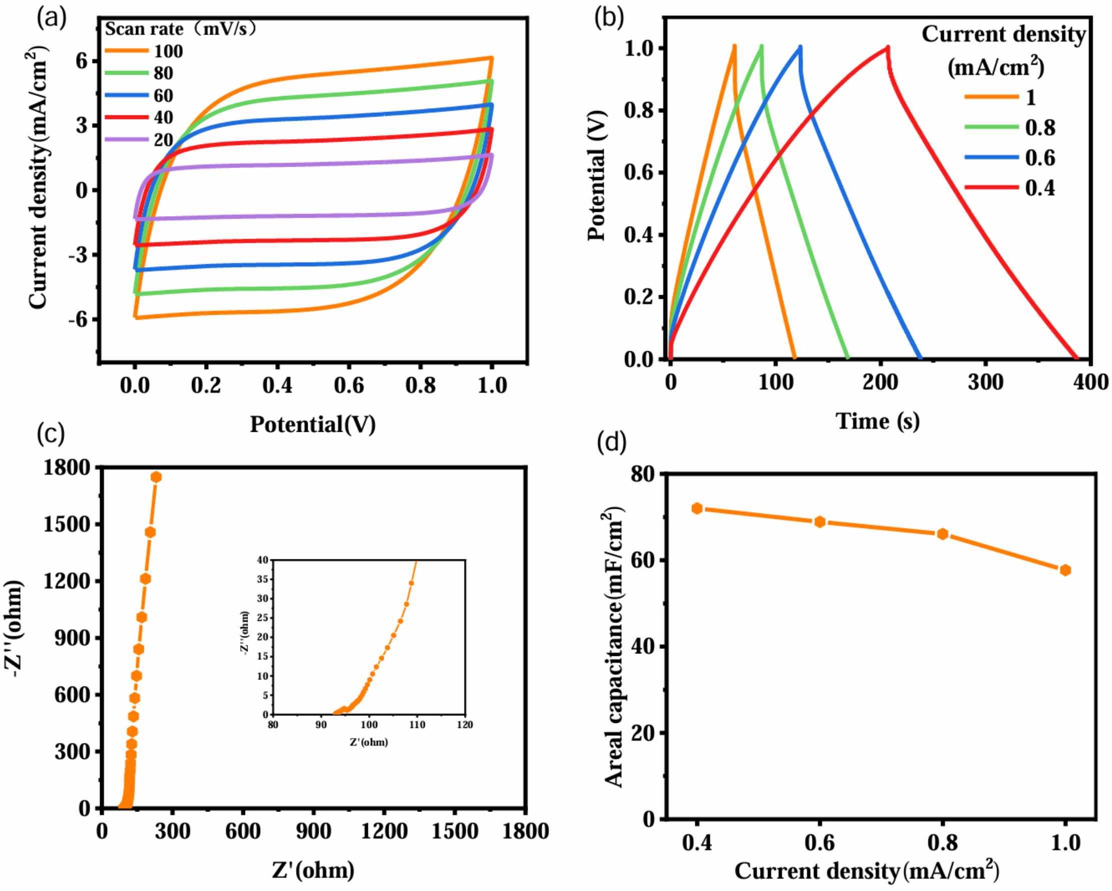

Figure S6. The electrochemical performance of symmetrical MSC based on PEDOT:PSS electrode. (a) CV curves at different scanning speeds; (b) GCD curves at different current densities; (c) Nyquist plot with an enlarged view in the insert; (d) Area capacitance at various current densities calculated from GCD curves.

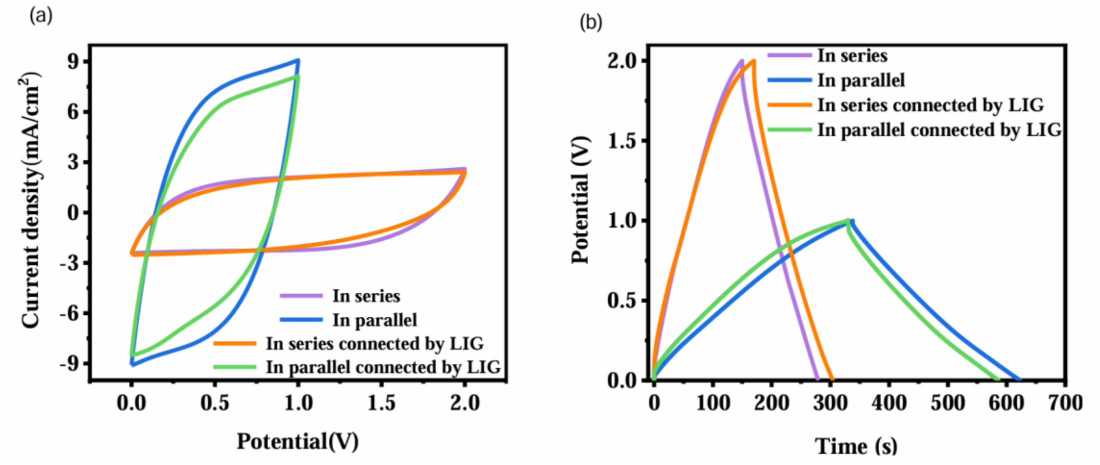

Figure S7. The comparation of LIGs and copper foil as connectors. (a) CV curves and (b) GCD curves of two AMSCs connected in series and parallel using copper foil and LIG respectively.

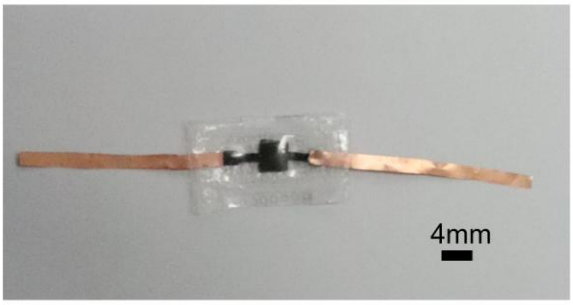

Figure S8. Optical photos of encapsulated AMSC.

Table S1. Comparison of electrochemical performance of LIG-AC//LIG-PEDOT:PSS with other LIG-based supercapacitors reported preciously.

Electrode	Electrolyte	Areal capacitance	Energy density	Power density	Retention	Ref
**LIG-AC//LIG-PEDOT:PSS**	**PVA/H_2SO_4**	**85.88 mF cm^{-2} at 0.4 mA cm^{-2}**	**11.93 μWh cm^{-2}**	**0.2 mW cm^{-2}**	**92.9%@10000, 91.6%@20000, 88.1%@50000**	**This work**
LIG	PVA/H_2SO_4	9.11 mF cm^{-2} at 0.01 mA cm^{-2}	/	/	98%@8000	[1]
a-LIG/MnO_2	PVA/ H_3PO_4	18.82 mF cm^{-2} at 0.2 mA cm^{-2}	2.61 μWh cm^{-2}	260.28 μW cm^{-2}	90.28%@ 5 000	[2]
AC-based LIG	KCl	20.7 mF cm^{-2} at 5 mV s^{-1}	/	/	/	[3]
LIG-N-PEDOT	PAAK/KOH	790 μF cm^{-2} at 50 μA cm^{-2}	/	/	/	[4]
LIG-C	H_3PO_4/PVA	2.33 mF cm^{-2} at 10 mV s^{-1}	/	/	102.4%@1000	[5]
MnO_2/LIG	PVA/H_2SO_4	48.9 mF cm^{-2}	3.1 μWh cm^{-2}	2.5 mW cm^{-2}	94.3%@3000	[6]
Al/AC//Al/lig–$NiWO_4$	PVA/H_3PO_4	17.01 mF cm^{-2} at 0.13 A g^{-1}	2 Wh cm^{-2}	100 W cm^{-2}	97%@1000	[7]
CS-LIG	PVA/KCl	15.1 mF cm^{-2} at 0.1 mA cm^{-2}	2.1 μWh cm^{-2}	50 μW cm^{-2}	80%@10000	[8]
LIG/AgNWs	PVP/NaCl	7.89 F cm^{-2} at 20 mV s^{-1}	9.87 μWh cm^{-2}	1.352 mW cm^{-2}	78%@1000	[9]

Activated carbon (AC), poly(3,4-ethylenedioxythiophene):poly(styrenesulfonate) (PEDOT:PSS), polyvinyl alcohol (PVA), laser-induced graphene (LIG), activated LIG (a-LIG), N-doped LIG absorbing PEDOT (LIG-N-PEDOT), potassium polyacrylate(PAAK), laser-induced MXene-graphene oxide composite (LIG-C), candle soot (CS), silver nanowires (AgNWs).

References

1. Peng, Z.; Lin, J.; Ye, R.; Samuel, E.L.G.; Tour, J.M. Flexible and Stackable Laser-Induced Graphene Supercapacitors. *ACS Applied Materials & Interfaces* **2015**, *7*, 3414-3419, doi:10.1021/am509065d.
2. Xi, S.; Gao, X.-W.; Cheng, X.-M.; Liu, H.-L. Deposition of MnO2 on KOH-

activated laser-produced graphene for a flexible planar micro-supercapacitor. *New Carbon Materials* **2023**, *38*, 913-924, doi:https://doi.org/10.1016/S1872-5805(23)60769-3.
3. Reina, M.; Scalia, A.; Auxilia, G.; Fontana, M.; Bella, F.; Ferrero, S.; Lamberti, A. Boosting Electric Double Layer Capacitance in Laser-Induced Graphene-Based Supercapacitors. *Advanced Sustainable Systems* **2022**, *6*, 2100228, doi:https://doi.org/10.1002/adsu.202100228.
4. Song, W.; Zhu, J.; Gan, B.; Zhao, S.; Wang, H.; Li, C.; Wang, J. Flexible, Stretchable, and Transparent Planar Microsupercapacitors Based on 3D Porous Laser-Induced Graphene. *Small* **2018**, *14*, 1702249, doi:https://doi.org/10.1002/smll.201702249.
5. Fu, X.-Y.; Shu, R.-Y.; Ma, C.-J.; Zhang, Y.-Y.; Jiang, H.-B.; Yao, M.-N. Self-assembled MXene-graphene oxide composite enhanced laser-induced graphene based electrodes towards conformal supercapacitor applications. *Applied Surface Science* **2023**, *631*, 157549, doi:https://doi.org/10.1016/j.apsusc.2023.157549.
6. Zhu, C.; Dong, X.; Mei, X.; Gao, M.; Wang, K.; Zhao, D. Direct laser writing of MnO2 decorated graphene as flexible supercapacitor electrodes. *Journal of Materials Science* **2020**, *55*, 17108-17119, doi:10.1007/s10853-020-05212-2.
7. Jha, S.; Mehta, S.; Chen, Y.; Renner, P.; Sankar, S.S.; Parkinson, D.; Kundu, S.; Liang, H. NiWO4 nanoparticle decorated lignin as electrodes for asymmetric flexible supercapacitors. *Journal of Materials Chemistry C* **2020**, *8*, 3418-3430, doi:10.1039/C9TC05811G.
8. Ghosh, A.; Kaur, S.; Verma, G.; Dolle, C.; Azmi, R.; Heissler, S.; Eggeler, Y.M.; Mondal, K.; Mager, D.; Gupta, A.; et al. Enhanced Performance of Laser-Induced Graphene Supercapacitors via Integration with Candle-Soot Nanoparticles. *ACS Applied Materials & Interfaces* **2024**, *16*, 40313-40325, doi:10.1021/acsami.4c07094.
9. Moradi, S.A.H.; Ghobadi, N.; Zahrabi, F. Highly conductive supercapacitor based on laser-induced graphene and silver nanowires. *Journal of Materials Science: Materials in Electronics* **2022**, *33*, 18356-18363, doi:10.1007/s10854-022-08690-z.
